# Study design of a phase 4, real-world study (COMPOSUR) to evaluate vibegron in patients with overactive bladder

**DOI:** 10.1186/s12894-023-01240-7

**Published:** 2023-04-24

**Authors:** Roger R. Dmochowski, Eric S. Rovner, Michael J. Kennelly, Diane K. Newman, Laleh Abedinzadeh, Daniel Snyder, Elizabeth Thomas, Cornelia Haag-Molkenteller, Matt T. Rosenberg

**Affiliations:** 1 Department of Urologic Surgery, Vanderbilt University Medical Center, 1211 Medical Center Dr, Nashville, TN 37232 USA; 2 Department of Urology, Medical University of South Carolina, Charleston, SC USA; 3 Carolinas Medical Center, Charlotte, NC USA; 4 Perelman School of Medicine, University of Pennsylvania, Philadelphia, PA USA; 5 Urovant Sciences, Irvine, CA USA; 6 Mid-Michigan Health Centers, Jackson, MI USA

**Keywords:** Adherence, Adrenergic beta-3 receptor agonists, Anticholinergic, Antimuscarinic, Medication persistence, Micturition, Telemedicine, Urinary bladder, Urinary incontinence

## Abstract

**Background:**

Overactive bladder (OAB) is defined as urinary urgency accompanied by frequency and nocturia, with or without urge urinary incontinence (UUI). Vibegron, a selective β_3_-adrenergic receptor agonist approved in the US in December 2020, demonstrated efficacy in reducing symptoms of OAB and was safe and well tolerated in the 12-week EMPOWUR trial and its 40-week, double-blind extension trial. The goal of the COMPOSUR study is to evaluate vibegron in a real-world setting to assess patient treatment satisfaction, tolerability, safety, duration of treatment, and persistence.

**Methods:**

This is a 12-month, prospective, observational, real-world study, with an optional 12-month extension to 24 months, in the US assessing adults ≥ 18 years old starting a new course of vibegron. Patients must be previously diagnosed with OAB with or without UUI, symptomatic for ≥ 3 months before enrollment, and receive prior treatment with an anticholinergic, with mirabegron, or with a combination of an anticholinergic and mirabegron. Enrollment is performed by the investigator following exclusion and inclusion criteria guided by US product labeling, reinforcing a real-world approach. Patients complete the OAB Satisfaction with Treatment Questionnaire (OAB-SAT-q) monthly and the OAB Questionnaire short form (OAB-q-SF) and Work Productivity and Activity Impairment Questionnaire (WPAI:US) at baseline and monthly for 12 months. Patients are followed up via phone call, in-person visits, or telehealth (ie, virtual) visits. The primary endpoint is patient treatment satisfaction as determined by the OAB-SAT-q satisfaction domain score. Secondary endpoints include percent positive responses to individual OAB-SAT-q questions, additional OAB-SAT-q domain scores, and safety. Exploratory endpoints include adherence and persistence.

**Discussion:**

OAB leads to a significant decrease in quality of life, as well as impairment of work activities and productivity. Persistence with OAB treatments can be challenging, often due to lack of efficacy and adverse effects. COMPOSUR is the first study to provide long-term, prospective, pragmatic treatment data for vibegron in the US and the resultant effect on quality of life among patients with OAB in a real-world clinical setting.

*Trial registration* ClinicalTrials.gov identifier: NCT05067478; registered: October 5, 2021.

## Background

Overactive bladder (OAB), defined as urinary urgency accompanied by frequency and nocturia, with or without urge urinary incontinence (UUI) [1], affects approximately 16.5% of adults in the US [2]. Women are more likely to have OAB with UUI vs without UUI, whereas men have a lower likelihood of OAB with UUI [2]. OAB is associated with bothersome symptoms that can negatively impact patient quality of life (QoL) [3, 4]. Patient assessments have shown that increased levels of symptom bother are associated with significant impairment in work productivity, as well as reduced health-related QoL (HRQL) [3, 4]. In patients with OAB, symptom bother is associated with reduced QoL due to depression and anxiety, the severity of which frequently correlates with level of symptom bother [5].

Patients with OAB and UUI have been shown to have significantly increased healthcare resource utilization (HCRU), including outpatient visits, diagnostic tests, and prescriptions filled, compared with those without OAB [6]. The economic burden of OAB is extensive; patients with OAB incur total healthcare costs exceeding 2.5 times costs compared with those without OAB in the US. This cost difference applies not only to OAB management but was also shown to be maintained across OAB-related comorbid conditions such as diabetes, depression, hypertension, and osteoporosis [7]. Further exacerbating HCRU burden, those treated with anticholinergics for OAB have a significantly higher risk of depression, anxiety, and falls/fractures [6].

Studies have highlighted low persistence rates with anticholinergics due to low treatment efficacy and adverse effects [8, 9]. Discontinuation rates for OAB pharmacotherapy can be up to 80% depending on the agent [10, 11], and rates have been shown to increase over time [12]. A systematic literature review reported 12-month persistence rates of approximately 22% with anticholinergic therapy [13]. On average, patients report > 2 reasons for discontinuation, which typically fall within 2 groups: (1) discontinuation due to lack of efficacy and adverse events and (2) discontinuation due to an aversion to taking any medications [9]. When asked about preference for OAB treatment options, patients place high importance on reduced daytime micturition frequency and nocturia and low side effects, including bladder-related side effects (urinary tract infection [UTI], dysuria, and urinary retention), dry mouth, and constipation [14].

Vibegron, a selective β_3_-adrenergic receptor agonist [15], demonstrated efficacy in reducing symptoms of OAB in the 12-week, international, phase 3, placebo- and active-controlled EMPOWUR trial [16] and its 40-week, double-blind extension trial [17]. In December 2020, vibegron was approved by the US Food and Drug Administration for the treatment of OAB in adults at a once-daily dose of 75 mg [18]. Currently, there are limited data showing how OAB drugs work in a real-world clinical setting; thus, we wanted to understand how a recently approved drug, vibegron, works in medical practice. Furthermore, the COVID-19 pandemic has altered the way medicine is practiced; however, utilization of telemedicine within urology practice due to the COVID-19 pandemic has shown patient satisfaction with urologic telemedical experiences [19]. This study uses telehealth visits to minimize in-person contact and allow for more visit flexibility during the COVID-19 pandemic. The goal of this study is to assess vibegron in real-world clinical practice in the US regarding patient treatment satisfaction, QoL, adherence, persistence, and safety.

## Methods

### Study design

**Fig. 1:**
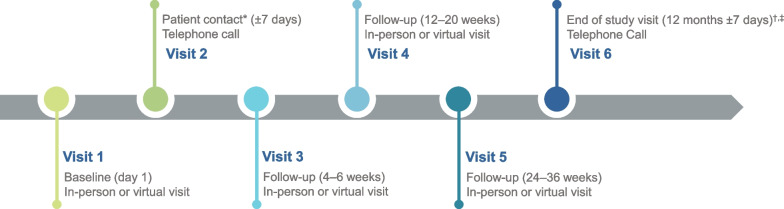
Schedule of baseline, follow-up, and final visits for enrolled patients. *Site staff virtual 2-week visit. ^†^Should the study sponsor extend the study an additional year, total per-patient follow-up will extend to 24 months. Additional in-person visits for OAB will occur for patients to receive their prescription refills 2 to 4 times in year 2 and to check for AEs around weeks 78 and 104. ^‡^Site staff virtual follow-up to record any new safety events. AE, adverse event; OAB, overactive bladder

COMPOSUR is a 12-month, prospective, observational, real-world study (NCT05067478), with an optional 12-month extension to 24 months, in patients with OAB at 60 study sites in the US. Study sites are principally urology and urogynecology but with some family medicine practices across academic centers and community practices. Study visits occur at week 2 and again at 4- to 6-week, 12- to 20-week, and/or 24-to 36-week intervals from baseline depending on the treating provider’s usual practice for routine follow-up of patients with OAB (Fig. 1), either in person or via telehealth, as well as at end of study or month 12. Treatments are prescribed and administered at the discretion of the treating provider in line with an observational, pragmatic approach and/or routine clinical practice. Patients are prescribed vibegron by the provider in accordance with US labeling for vibegron and receive their prescriptions at a pharmacy.

This study is being conducted in compliance with the Declaration of Helsinki and with International Council on Harmonisation of Technical Requirements for Pharmaceuticals for Human Use/Good Clinical Practice guidelines. Investigators receive institutional review board (IRB) approval via central and/or local IRB, research ethics board approval, or independent ethics committee approval before the initiation of the study. All patients provide written informed consent.

### Study population

Key inclusion and exclusion criteria are presented in Table 1. Briefly, the study includes men and women ≥ 18 years of age at baseline with a diagnosis of OAB with or without UUI, with symptoms for ≥ 3 months prior to baseline visit, initiating a new course of treatment with vibegron in relation to this study, able and willing to complete electronic patient-reported outcome questionnaires monthly for a minimum of 1 year, previous exposure to mirabegron monotherapy and/or mirabegron plus solifenacin and/or anticholinergics prior to initiation of vibegron, and current commercial or government insurance prescription drug coverage.

**Table 1:** Enrollment criteria of the COMPOSUR study

Inclusion criteria	Exclusion criteria
Male or female, ≥ 18 years of age	Any contradiction to the use of vibegron per US label
Able to provide informed consent	History of mixed incontinence where stress incontinence is the predominant form (as determined by the investigator)
Diagnosis of OAB with or without UUI by the treating healthcare provider or previous healthcare provider	Patients at risk of urinary retention (as determined by the investigator)
Symptoms of OAB ≥ 3 months before baseline visit	Neurologic conditions associated with OAB symptoms (eg, multiple sclerosis)
Initiating of new course of vibegron and able to complete electronic PRO questionnaires monthly for a minimum of 1 year	Pregnant or breastfeeding or plans to do so during the study
Previous exposure to anticholinergics or mirabegron monotherapy and/or mirabegron plus solifenacin combination	Participation in another clinical trial with investigational product or device
Commercial or government-issued insurance coverage	History of OAB treatment with botulinum toxin A, SNM, PTNS, external beam radiation therapy, or urinary stents ≤ 6 months; pelvic or lower urinary tract surgery ≤ 6 months; and urethral catheterizations ≤ 3 months before baseline visit
	Use of vibegron before baseline visit either prescribed or in a previous vibegron clinical trial where patient was on vibegron
	Anyone who, at investigator discretion, is not suitable for treatment with a β_3_-adrenergic agonist for OAB for any reason

OAB, overactive bladder; PRO, patient-reported outcome; PTNS, percutaneous tibial nerve stimulation; SNM, sacral neuromodulation; UUI, urge urinary incontinence

Exclusion criteria include any history of OAB treatment with botulinum toxin A, sacral neuromodulation, percutaneous tibial nerve stimulation, external beam radiation therapy, or urinary stents within the last 6 months; pelvic or lower urinary tract surgery within the last 6 months; or urethral catheterizations within the last 3 months. Additional exclusion criteria are a history of mixed incontinence (ie, OAB with UUI and stress incontinence) where the investigator determined that stress incontinence was predominant, patients at risk for urinary retention as determined by investigator, neurologic conditions associated with OAB (eg, multiple sclerosis), pregnant or breastfeeding or plans to do so during study, and prior use of vibegron before baseline visit.

### Study assessments

Visit 1 (baseline) is conducted either as an in-person or virtual visit, during which study staff acquire patient demographics, current measures of health, medical and OAB history including reasons for discontinuing prior OAB medication, and overall prior and concomitant medications in the past 4 months at the baseline visit (Fig. 1). A follow-up phone call is conducted for the second visit (week 2) to collect information pertaining to adverse events (AEs), concomitant medication use, and pregnancy status for female patients of child-bearing potential. During each subsequent follow-up visit (visits 3, 4, and 5), performed either in person or via telehealth, the study coordinator will ask the study questions and enter responses into the electronic data capture system. Patients complete the OAB Satisfaction with Treatment Questionnaire (OAB-SAT-q; Table 2) monthly for 12 months. Additionally, at baseline and monthly for 12 months, patients complete the OAB Questionnaire short form (OAB-q-SF) covering symptom bother and HRQL; the Work Productivity and Activity Impairment Questionnaire‒Urinary Symptoms (WPAI:US); and other questions related to incontinence pad use, OAB provider visits, UTIs (based on number in past 6 months for baseline and number in past month for monthly visits), nocturia, and hours of uninterrupted sleep (Table 2). Patients can access the questionnaires online every month. The OAB-SAT-q is an 11-item questionnaire that assesses patients’ satisfaction with their OAB treatment within a clinical setting and includes 3 subscales (satisfaction, side effects, endorsement) and 2 single-item assessments (convenience, preference) using a 4-week recall period [20]. Scoring ranges from 0‒100 for domains and the convenience item, whereas the preference item is calculated as a percentage. Higher scores indicate higher levels of treatment satisfaction, endorsement, and convenience. The OAB-q-SF evaluates bladder symptoms (6 questions) and their impact on HRQL (13 questions) and is scored from 0‒100, with higher scores indicating increased bother and reduced HRQL [21]. The WPAI:US is a 6-question form that evaluates active employment, the number of work hours missed and lost productivity over the past 7 days, and the effect of OAB on ability to do regular activities [22]. Safety, consisting of AEs, are recorded throughout the study; additionally, the last visit is conducted via telephone as the final check on patient safety for the study.

**Table 2:** Schedule of baseline and follow-up assessments

Assessment	Baseline (day 1)	Follow-up (monthly*)
Pregnancy status	X	X
Concomitant medications^†^	X	X
Details of new course of vibegron^‡^	X	X
Medication compliance question	X	X
OAB-q-SF	X	X
OAB-SAT-q		X
WPAI:US	X	X
Incontinence pad use over last 7 days	X	X
Provider visits for OAB	X	X
UTIs	X	X
Nocturia episodes	X	X
Hours of uninterrupted sleep before waking to use restroom	X	X

OAB, overactive bladder; OAB-q-SF, Overactive Bladder Questionnaire-short form; OAB-SAT-q, Overactive Bladder Satisfaction questionnaire; UTI, urinary tract infection; WPAI:US, Work Productivity and Activity Impairment‒urinary symptoms

^*^Visit window, ± 7 days. Visits 1, 3, 4, and 5 are in person or via telehealth; visits 2 and 6 are phone calls

^†^Taking any new medication and reason for discontinuing any other medications

^‡^Still on vibegron and reason for discontinuation of vibegron if stopped

### Endpoints

Endpoints are evaluated at 3-, 6-, and 12-month intervals from baseline. The primary endpoint is patient treatment satisfaction at month 12 as determined by the OAB-SAT-q satisfaction domain score. Key secondary endpoints supporting the primary endpoint include percent positive responses to individual satisfaction questions 1, 2, 3, and 11. Additional secondary endpoints include OAB-SAT-q scores for side effects, endorsement, convenience, and preference and reasons for treatment discontinuation. Exploratory endpoints include persistence and adherence. Safety will be assessed by types and incidence of AEs.

### Statistical analysis

The full analysis set consists of all enrolled patients who receive ≥ 1 dose of vibegron and have ≥ 1 postbaseline assessment and is used for the analysis of treatment satisfaction, QoL, HCRU, and treatment patterns. The safety analysis set consists of all enrolled patients who receive ≥ 1 dose of vibegron and is used for analysis of safety data. Patients are assigned to 1 of 2 cohorts based on previous OAB treatment exposure: Cohort A consists of patients with previous anticholinergic therapy, whereas cohort B consists of patients with previous mirabegron monotherapy or combination therapy with solifenacin. Summaries will be reported for each cohort separately and for the combined cohorts.

Descriptive analyses will be performed to assess quantitative data collected to understand the characteristics of the cohorts studied. Interim analyses will be conducted after most patients have completed the 3- and 6-month questionnaires; the final analysis will be performed 12 months after the last patient is enrolled. Data will be analyzed using SAS^®^ version 9.2 or higher (SAS Institute, Cary, NC, USA). A sample size of 400 enrolled patients (≥ 100 patients per cohort) will provide for a 95% confidence interval with lower and upper bounds within ± 3.635 of the mean.

## Discussion

This 12-month observational study, with an optional 12-month extension, will be conducted in a US practice setting to gain real-world insights regarding the impact of vibegron for the treatment of OAB on patient treatment satisfaction, QoL, HCRU, work productivity, safety and tolerability. To better reflect the real-world approach of the study, inclusion and exclusion criteria are broad and are based on the US product label for vibegron to better represent patient selection in clinical practice. Study visits will be conducted by patients’ providers as part of their usual practice, and study endpoints focus on patient-reported outcomes.

Randomized controlled trials (RCTs) are key for determining the efficacy of a drug and are complemented by real-world evidence, which assesses the overall effectiveness of a drug while presenting an opportunity to examine outcomes that are difficult to measure in clinical trials such as HCRU [23, 24]. Although RCTs can control for confounding, selection bias, and information bias, the inherent selection criteria may not be fully generalizable to the general population [23, 24]. Advances in methodologies and statistical analysis have increased the quality of real-world evidence closer to the level expected in RCTs, resulting in the data gathered from real-world trials being additive and complementary to data gathered in RCTs [24].

Previous real-world, observational studies of pharmacotherapy for OAB have used methodologies similar to that employed in this protocol. To evaluate patient perception of efficacy and overall treatment satisfaction, COMPOSUR uses the OAB-q-SF questionnaire, assessing system bother and HRQL, which has also been used in a previous real-world study [25]. Although treatment satisfaction is sometimes assessed with the single-question Treatment Satisfaction Visual Analog Scale [25, 26], COMPOSUR uses the OAB-SAT-q to measure patient satisfaction with treatment, enabling greater assessment of not only satisfaction but also side effects, convenience, and preference. COMPOSUR will monitor patients over a 12-month period, with patient visits occurring slightly more frequently than a previous 12-month-long real-world study [25]; however, frequency of study visits is representative of usual practice for treating providers. A strength of COMPOSUR over similar studies of shorter duration [26, 27] is the ability to capture a longer time span of data, which is relevant given the chronic nature of OAB. Similar to other trials [25, 26], enrollment criteria include patients who had previously been on pharmacotherapy for OAB in order to reflect past treatment experience and expectations.

Leveraging telemedicine is a unique component of this OAB study as use of telemedicine by urologists and urogynecologists has roughly tripled owing to the COVID-19 pandemic [28], a rate that exceeds what has been seen in primary care visits [29]. In urology-related subspecialties it has been shown that telehealth use results in effective patient encounters [19]. Patients and providers report being satisfied with telehealth encounters [30], and by increasing provider access, appointment wait times are reduced [31]. Combined with phone calls and in-person visits, telehealth visits provide additional convenience for patients while unburdening healthcare delivery systems, further strengthening this method of care delivery.

Adherence and persistence with OAB treatments can be challenging, and persistence frequently decreases over time, with 12-month persistence rates ranging from 20 to 69% [8, 10, 12]. A real-world study evaluating claims data showed rates of 12-month treatment failure, defined as either treatment discontinuation or treatment switching, of ≥ 80% for both mirabegron and anticholinergics [32]. Medication nonadherence may be intentional, commonly due to adverse effects [33] and questionable cost benefit for the patient, further complicating persistence rates. The nature of low morbidity in OAB has been suggested to lower the persistence threshold, with the most frequent discontinuation reasons being lack of efficacy and adverse effects [8, 9, 12]. Although there have been concerns about β_3_-adrenergic agonists elevating blood pressure, treatment with vibegron has been shown to have no statistically significant or clinically meaningful impact on blood pressure compared with placebo [34], which has the potential to lead to improved persistence.

Studies using the OAB-q and WPAI:US have shown that patients with OAB experience significantly decreased work productivity and HRQL, as well as impairment in daily activities, with significantly increased HCRU [3, 4, 35]. Vibegron has been shown to significantly improve QoL in patients with OAB in a clinically meaningful manner as reported by patients [36, 37]. Improving QoL by mitigating bothersome symptoms of OAB in a real-world setting is a key component of COMPOSUR.

This study design is subject to limitations inherent to open-label, real-world studies including lack of randomization and absence of a control group. There is potential for selection bias (ie, enrollment bias) as patients who enroll in the study may differ from patients who do not enroll. Because patients will have tried and failed other oral OAB treatments, there is the potential for implicit bias. Future studies may be needed to assess these outcomes in subsets of patients, including a socioeconomically disadvantaged population and residents of long-term care and assisted living facilities. Results will only be compared to a patient’s own baseline values; thus, patient recall may be limited in some of the baseline values that have longer look backs.

In conclusion, our study will be the first to provide long-term, prospective treatment satisfaction and persistence data for vibegron in the US among patients with OAB who received prior treatment with an anticholinergic or with mirabegron. The pragmatic, observational aspect of patients who are representative of real-world vibegron users will provide greater insight into real-world use and outcomes.

## Data Availability

Not applicable.
